# Impact of AI on Breast Cancer Detection Rates in Mammography by Radiologists of Varying Experience Levels in Singapore: Preliminary Comparative Study

**DOI:** 10.2196/66931

**Published:** 2025-11-24

**Authors:** Serene Si Ning Goh, Hao Du, Loon Ying Tan, Edward Zhen Yu Seah, Wai Keat Lau, Alvin Hong Zhi Ng, Shi Wei Desmond Lim, Han Yang Ong, Samuel Lau, Yi Liang Tan, Mun sze Khaw, Chee Woei Yap, Kei Yiu Douglas Hui, Wei Chuan Tan, Haziz Siti Rozana Binti Abdul, Vanessa Mei Hui Khoo, Shuliang Ge, Felicity Jane Pool, Yun Song Choo, Yi Wang, Pooja Jagmohan, Premilla Pillay Gopinathan, Mikael Hartman, Mengling Feng

**Affiliations:** 1 Saw Swee Hock School of Public Health, National University Health System, National University Hospital Singapore, 12 Science Drive 2, Singapore, 117549, Singapore, 65 6516 4984; 2 Department of Breast and General Surgery, National University Health System, Singapore, Singapore; 3 Yong Yoo Lin School of Medicine, National University of Singapore, Singapore, Singapore; 4 Department of Diagnostic Imaging, National University Hospital, Singapore, Singapore; 5 Institute of Data Science, National University of Singapore, Singapore, Singapore

**Keywords:** mammography, multi-reader, multi-case, detection, breast cancer, mammograms, radiologist, radiology, Singapore, AI, artificial intelligence, cancer, women, diagnosis, diagnostic modality

## Abstract

**Background:**

Breast cancer remains the most common cancer among women globally. Mammography is a key diagnostic modality; however, interpretation is increasingly challenged by rising imaging volumes, a global shortage of breast radiologists, and variability in reader experience. Artificial intelligence (AI) has been proposed as a potential adjunct to address these issues, particularly in settings with high breast density, such as Asian populations. This study aimed to evaluate the impact of AI assistance on mammographic diagnostic performance among resident and consultant radiologists in Singapore.

**Objective:**

To assess whether AI assistance improves diagnostic accuracy in mammographic breast cancer detection across radiologists with varying levels of experience.

**Methods:**

A multi-reader, multi-case study was conducted at the National University Hospital, Singapore, from May to August 2023. De-identified digital mammograms from 500 women (250 with cancer and 250 normal or benign) were interpreted by 17 radiologists (4 consultants, 4 senior residents, and 9 junior residents). Each radiologist read all cases over 2 reading sessions: one without AI assistance and another with AI assistance, separated by a 1-month washout period. The AI system (FxMammo) provided heatmaps and malignancy risk scores to support decision-making. Area under the curve of the receiver operating characteristic (AUROC) was used to evaluate diagnostic performance.

**Results:**

Among the 500 cases, 250 were malignant and 250 were non-malignant. Of the malignant cases, 16%(80/500) were ductal carcinoma in situ and 84%(420/500) were invasive cancers. Among non-malignant cases, 69.2%(346/500) were normal, 17.6%(88) benign, and 3.6%(18/500) possibly benign but stable on follow-up. Masses (54.4%, 272) and calcifications (10.8%, 54/500) were the most common findings in cancer cases. A majority of both malignant (66.8%, 334/500) and non-malignant (68%, 340/500) cases had heterogeneously or extremely dense breasts (BIRADS [Breast Imaging Reporting and Data System] categories C and D). The AI model achieved an AUROC of 0.93 (95% CI 0.91‐0.95), slightly higher than consultant radiologists (AUROC 0.90, 95% CI 0.89‐0.92; *P*=.21). With AI assistance, AUROC improved among junior residents (from 0.84 to 0.86; *P*=.38) and senior residents (from 0.85 to 0.88; *P*=.13), with senior residents approaching consultant-level performance (AUROC difference 0.02; *P*=.051). Diagnostic gains with AI were greatest in women with dense breasts and among less experienced radiologists. AI also improved inter-reader agreement and time efficiency, particularly in benign or normal cases.

**Conclusions:**

This is the first study in Asia to evaluate AI assistance in mammography interpretation by radiologists of varying experience. AI significantly improved diagnostic performance and efficiency among residents, helping to narrow the experience-performance gap without compromising specificity. These findings suggest a role for AI in enhancing diagnostic consistency, improving workflow, and supporting training. Integration into clinical and educational settings may offer scalable benefits, though careful attention to threshold calibration, feedback loops, and real-world validation remains essential. Further studies in routine screening settings are needed to confirm generalizability and cost-effectiveness.

## Introduction

Mammograms are a critical tool in breast cancer diagnosis; however, interpreting them is inherently challenging. Expertise is acquired only after lengthy training; however, there is a shortage of seasoned senior radiologists [1] due to workforce aging and rising demand for breast cancer screening and diagnosis. The scarcity of skilled professionals in this field is particularly critical in health care systems that increasingly prioritize health screening and primary prevention. The challenge in the interpretation of mammography is further confounded when faced with dense breasts, as higher breast densities tend to obscure detection of breast lesions, a unique problem among Asian women compared to their Western counterparts [2]. The introduction of artificial intelligence (AI) presents a potential solution to this issue. In recent years, there has been a growing interest in leveraging AI in medical imaging, particularly with the development and application of deep learning algorithms for digital mammography [3,4]. Preliminary investigations indicate that AI systems, when used as concurrent readers for mammogram interpretation, can enhance radiologist efficiency in terms of time, sensitivity, and specificity [5,6].

Beyond the potential workload reduction and improved cancer detection, AI features such as heatmaps and triage capabilities may provide valuable training support for resident radiologists.

Interest in AI among residents is growing, especially after its inclusion in the noninterpretive skills section of the Qualifying (Core) Exam by the American Board of Radiology in 2021 [7]. The integration of AI into residency clinical workflows may extend beyond its potential role in reducing diagnostic errors; it has the potential to offer continuous mentorship, especially during times when consultants’ expertise may not be readily available. This creates a supportive and learning-oriented work environment for resident radiologists. AI could also serve as a tool for personalized precision education [8], enabling each resident to accumulate expertise and receive tailored feedback to enhance their performance. As AI becomes an integral part of residency curriculums, working with it will be a crucial non-interpretive skill that residents must acquire. Looking ahead, residents will not only interpret mammographic images but also need to be attuned to unusual AI outputs, recognize and address automation bias, and understand how AI can alter clinical workflows [9]. Furthermore, AI can be trained to triage benign cases, reducing the burden of routine tasks and allowing radiologists to focus on higher-value responsibilities such as analyzing complex cases. It has the potential to enhance efficiency and mitigate the risk of burnout [10].

Despite the aforementioned benefits, there remains a paucity of studies evaluating the performance of AI algorithms for mammograms in the context of assisting resident radiologists. Therefore, this study is designed to investigate the performance of AI assistance for resident radiologists compared to consultant radiologists for breast cancer detection.

## Methods

### Ethical Considerations

The research protocol received approval from the National Health Group Institutional Review Boards on December 16, 2022 (reference number 2022/00843). Informed consent was waived due to the use of de-identified, retrospective imaging data.

### Case Selection

A multi-reader multi-case (MRMC) investigation was conducted at the National University Hospital (NUH) from May to August 2023. The study involved a retrospective analysis of de-identified mammographic images obtained from the institutional radiology archive. All available mammographic examinations, including both screening and diagnostic mammograms conducted during the study period, were considered. Cases were identified through a systematic review of radiology reports and corresponding pathology records to ensure diagnostic confirmation and completeness. The inclusion criteria were (1) women aged 40 years and older; (2) presence of 4 standard views on mammograms; (3) availability of biopsy-proven results for malignant cases; and (4) absence of a breast cancer diagnosis after a 24-month follow-up for normal cases. Cases were excluded if they exhibited any of the following conditions: (1) evidence of mammographic needle projection, pre-biopsy markings, or clips; (2) mammographic artifacts from breast implants; (3) poor-quality mammograms; (4) malignant cases lacking corresponding biopsy-proven histology; and (5) malignant cases with histology reports exceeding 3 months from the mammogram date to ensure that cancers included were promptly detected following the mammograms. The accuracy of malignant cases was verified through biopsy results. Non-malignant cases included Breast Imaging Reporting and Data System (BIRADS) 1 normal mammogram, BIRADS 2 (benign) and BIRADS 3 (possible benign) lesions on mammograms were either biopsy-proven benign or confirmed by the absence of a breast cancer diagnosis during a 24-month follow-up period. To ensure balanced representation and maintain feasibility for a multi-reader design, the dataset was enriched to include an equal number of cancer (n=250) and non-cancer (n=250) cases, reflecting a 1:1 ratio. This approach, commonly used in diagnostic reader studies, aimed to maximize statistical power while maintaining case diversity. All images were fully anonymized prior to distribution for interpretation, and no patient-identifiable information was accessible to readers or study investigators.

### Sample Size Calculation

This retrospective, MRMC study was conducted with 500 cases and 17 radiologist readers. An equal sample of malignant cases and non-malignant cases produces for any individual reader a 2-sided 95% CI for the area under the curve of the receiver operating characteristic (AUROC) with a width (difference between lower and upper limit) no wider than 0.227. The same level of precision will be provided for specificity, as there are 250 non-malignant cases. The reader study image case set was enriched with cancer cases by having a 1:1 normal-to-malignant case ratio. This enriched case set provides an efficient and less burdensome representative case dataset. Hillis and Schartz [11] recommended “that a researcher use at least 4 readers, and preferably more, if the goal is to generalize to both reader and case populations.” As the variability between consultant and senior resident radiologists is expected to be smaller compared to variability between resident radiologists, 4 consultants, 4 senior radiologists, and 9 resident radiologists would provide reasonably precise estimates of reader variability to prove the hypothesis.

### Reader Characteristics

In total, 17 radiologists from NUH participated in the study, comprising 4 consultants, 4 senior residents, and 9 junior resident radiologists. Resident radiologists were classified as radiology trainees who had completed the Fellowship of the Royal College of Radiologists examination and were undergoing training as part of The Accreditation Council for Graduate Medical Education radiology residency programs during the study period [12,13]. Senior resident radiologists were in their final year of radiology residency, while junior resident radiologists were in Years 3 and 4 of the program. Consultant radiologists were defined as those who had received accreditation from the specialty of Diagnostic Radiology of the Specialists Accreditation Board in Singapore [14]. None had prior experience of reading mammograms with AI assistance in a study trial or clinical setting at the time of the study.

### AI Software

All examinations were retrospectively processed using FxMammo (version 1.1.0; FathomX) [15], which has received Health Science Authority approval from Singapore. The AI system generates a continuous risk score for each view of both sides of the breast, ranging from 0% to 100%, with higher scores indicating a greater risk of suspicious findings. The highest risk score from each mammography examination was used to determine the overall patient-level risk score. The system is able to provide varying cutoff thresholds for malignancy score, which defines the levels at which the algorithm classifies an image as positive for malignant breast cancer. The reference cutoff for malignancy in this study was determined by the Youden index [16]. The results are presented to the clinician as a “heatmap,” highlighting the regions of interest on the mammogram with their corresponding risk scores.

### Study Protocol

All radiologists read unique mammograms, acquired using the same mammographic system, of 500 women over a 6-week period without AI assistance. This was followed by a 1-month washout period and then another 6 weeks of reading the same mammograms with AI assistance. The order of the mammograms was shuffled in the second reading. All radiologists underwent a pilot trial before the study commenced, during which they were familiarized with the reading process on the OsiriX DICOM Viewer [17] and the recording of their interpretations on an electronic form. The form required recording of the lesion site for suspected malignant cases (left or right breast), malignancy risk score (yes or no), and BIRADS density assessment. Readers were not informed of the cancer enrichment in the dataset. Varying cutoff thresholds of the AI, including the threshold at Youden index for malignancy score, were provided to readers so that they are able to balance their sensitivity and specificity by adjusting the criteria for classifying cases. The time taken to read each case, encompassing interpretation and form completion, was recorded by a research assistant.

### Data Analysis

Statistical analysis was performed using Python (version 3.8.16) and R (version 3.6.1, package RJafroc version 2.1.2, pROC version 1.18.4). Radiologists were grouped by their reader experience levels into junior residents, senior residents, and consultant radiologists. Sensitivity, specificity, and accuracy of malignancy risk scores were calculated in percentages. The difference in proportions between the various diagnostic metrics before and after AI assistance was compared using 2-tailed permutation tests with 2000 iterations [18,19]. AUROCs were calculated for each group of radiologists for malignancy risk scores assigned to each case compared to the ground truth with and without AI assistance. AUROCs were reported for each group of radiologists under each modality (eg, with or without AI assistance). For each group of radiologists, the non-parametric (trapezoidal) AUROC for the “with AI” read, “without AI” read, and the difference between them was presented. The comparison of AUROC performance in the MRMC analysis was conducted using the Obuchowski-Rockette-Hillis method [20,21] and DeLong’s test [22]. Covariance terms in the model were estimated using the jackknife resampling method. The jackknife method was used to estimate covariance terms in the model. Two‐sided 95% CIs were used to illustrate the precision of the within‐modality estimates and the between‐modalities difference. Inter-reader agreement in assessing cancer detection was determined using percent agreement and Cohen kappa (*k*). A value of *P*<.05 was considered statistically significant. To account for multiple comparisons, including AUROC, accuracy, sensitivity, specificity, and Cohen kappa, the significance level was adjusted to 0.01 using the Bonferroni correction [23]. Two-tailed paired t-tests were used to compare time savings before and after assistance.

### Outcomes

The primary objective of the study is to compare diagnostic accuracy in terms of cancer detection (binary outcome, yes or no cancer) between resident and consultant radiologists with and without AI assistance, measured by AUROCs. Secondary endpoints include the time taken to read each case.

## Results

### Case Characteristics and Histology

A total of 500 women with 2000 mammographic images were eligible, including 250 non-malignant cases and 250 malignant cases (Table 1). The median age of women with malignancy was 60.2 years (IQR: 51.8-68.0) as compared to 53.0 years (IQR: 47.0 to 60.0) for women with no malignancy (*P*<.001). Of the malignant cases, 16% were ductal carcinoma in situ and 84% were invasive cancer. Of the non-malignant cases, 69.2% had normal mammograms, 17.6% had benign lesions, and 3.6% had possibly benign lesions that completed 24 months of normal follow-up. Mass (54.4%) and calcification (10.8%) were the most common lesion types for malignant cases. The majority of non-malignant cases (68%) and malignant cases (66.8%) had BIRADS density categories of C and D.

**Table 1.** Summary of clinical and histopathological characteristics of all included cases.

Characteristics	Non-malignant (n=250)	Malignant (n=250)	*P* value
Age of women (median, IQR)	53.0 (47.0, 60.0)	60.2 (51.8, 68.0)	<.001^a^
BIRADS^b^ density category, n (%)			
A	8 (3.2)	7 (2.8)	.496
B	72 (28.8)	76 (30.4)	
C	151 (60.4)	156 (62.4)	
D	19 (7.6)	11 (4.4)	
Lesion type category, n (%)			
Mass	15 (10.1)	136 (54.4)	.001^a^
Calcification	12 (8.1)	27 (10.8)	
Asymmetry	98 (65.8)	21 (8.4)	
Distortion	6 (4.0)	7 (2.8)	
Mass+ calcification	2 (1.3)	27 (10.8)	
Combined lesions	16 (10.7)	32 (12.8)	
BIRADS malignancy category and histopathology type category, n (%)			
Normal mammograms (BIRADS 1)	173 (69.2)	1 (0.4)	.001^a^
Benign (BIRADS 2)	44 (17.6)	0 (0)	NA
Possibly benign (BIRADS 3)	9 (3.6)	1 (0.4)	.001^a^
Suspicious (BIRADS 4)	(Biopsy proven benign and concordant) 24 (9.6)	160 (64.0)	.001^a^
Highly suggestive of malignancy (BIRADS 5)	0 (0)	88 (35.2)	NA

a Statistically significant with *P* value of <.05.

b BIRADS: Breast Imaging Reporting and Data System.

### Comparison of Cancer Diagnostic Performance by Reader Experience Before and After AI Assistance

**Figure 1.**
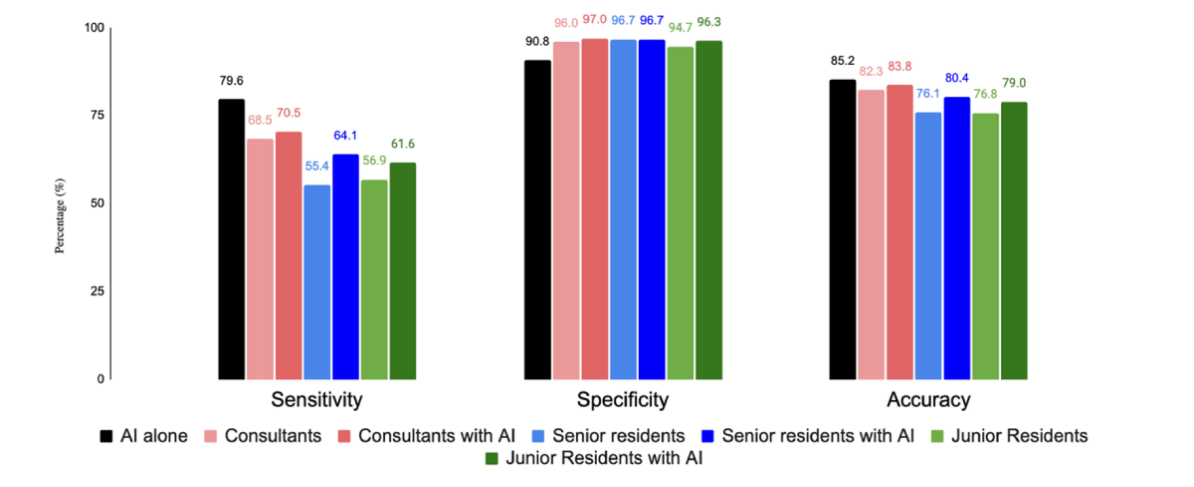
Sensitivity, specificity, and accuracy grouped by reader experience without and with artificial intelligence (AI) assistance.

Reading with AI assistance improved the sensitivity of junior resident radiologists from 56.9% to 61.6% (difference 4.7%, 95% CI difference [1.9, 7.1% ], *P*<.001) and senior resident radiologists from 55.4% to 64.1% (difference 8.7 %, 95% CI difference (4.5%, 13%) *P*<.001) (Figure 1). Reading with AI assistance improved the sensitivity of consultant radiologists from 68.5% to 70.5% (difference 2%, 95% CI difference (−1.9%, 6.4%), *P*=.35), respectively. Similarly, with AI assistance, an improved specificity was observed among junior resident radiologists (94.6%-96.3%; (difference 1.7%, 95% CI difference (0.4%, 2.9%), *P*=.02), senior resident radiologists (96.7%-96.7%; difference 0%, 95% CI difference (−1.6%, 1.5%), *P*=.99), and consultant radiologists (96%-97%; difference 1%, 95% CI difference (−0.6%, 2.6%), *P*=.22) as compared with reading without AI assistance. Junior resident radiologists had improved accuracy from 75.8% to 78.9% (difference 3.1%, 95% CI of difference (1.3%, 4.9%), *P*=.005) and senior resident radiologists had improved accuracy from 76.1% to 80.4% (difference 4.3%, 95% CI of difference (1.8%, 6.9%), *P*=.002) Consultant radiologists had improved accuracy from 82.3% to 83.9% (difference 1.6%, 95% CI of difference (0.8%, 3.8%) , *P*=.24).

### Comparison of AUROC by Reader Experience Before and After AI Assistance

Use of the AI system standalone demonstrated an AUROC of 0.93 (95% CI 0.91-0.95), as compared to 0.90 (95% CI 0.89‐0.92) for consultant radiologists, 0.85 (95% CI 0.83-0.87) for senior resident radiologists, and 0.84 (95% CI (0.83-0.85) for junior resident radiologists.

**Figure 2.**
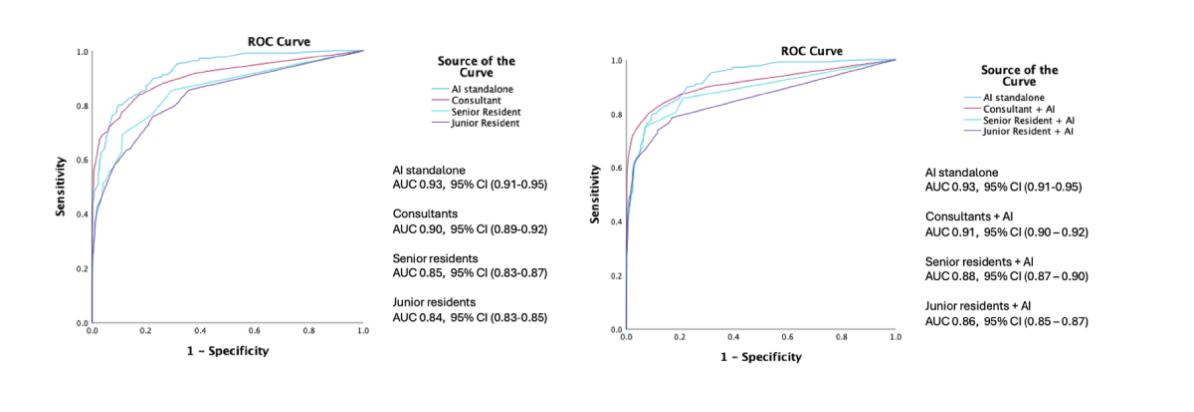
Comparison of area under the curve of the receiver operating characteristic (AUROC) for junior and senior resident radiologists with and without artificial intelligence (AI) assistance.

The diagnostic performance of AI standalone is significantly higher than that of consultant radiologists (difference in AUROC 0.02, 95% CI of difference –0.01, 0.05, *P*=.21) (Figure 2). AI’s diagnostic performance was higher than both senior (difference in AUROC 0.06, 95% CI of difference 0.02, 0.11 *P*=.013) and junior residents’ (difference in AUROC 0.07, 95% CI of difference 0.05, 0.10*P*<.001) assessments, respectively. There is no statistically significant difference between senior and junior residents’ performance (difference in AUROC 0.006, 95% CI of difference −0.013, 0.026, *P*=.52).

With AI assistance, both junior and senior residents showed improvement in diagnostic performance, AUROC from 0.85 to 0.86 (difference 0.008, 95% CI of difference –0.011, 0.027, *P*=.38), and AUROC 0.87 to 0.89 (difference 0.027, 95% CI of difference –0.014, 0.068, *P*=.13), respectively. With AI assistance, the AUROC of senior residents was comparable to consultant radiologists with a difference in AUROC of 0.02 (95% CI 0.00‐0.039, *P*=.051). However, the diagnostic performance of junior residents remained lower than consultant radiologists despite AI assistance (difference in AUROC 0.045, 95% CI of difference 0.028, 0.061, *P*<.001).

The AUROCs were lower for all reader groups when interpreting mammograms of women with high breast density compared to those with non-dense breasts, at 0.82 (95% CI 0.81‐0.83) and 0.93 (95% CI 0.92‐0.93), respectively. However, this difference was not statistically significant (*P*=.20). With AI assistance, the AUROC for women with dense breasts improved to 0.84 (95% CI 0.83‐0.85) from 0.82 (95% CI 0.81‐0.83), although the increase was not statistically significant (*P*=.13). Similar improvements were observed across all other reader groups, but none reached statistical significance (Table 2).

**Table 2.** Comparison of AUROC^a^ for reader groups based on breast density with and without AI^b^ assistance.

Breast density	AUROC of reader groups without AI assistance (95% CI)				AUROC of reader groups with AI assistance (95% CI)			
	All	Consultants	Senior residents	Junior residents	All	Consultants	Senior residents	Junior residents
Non-dense breast group (density A and B)	0.93 (0.92‐0.93)	0.96 (0.94‐0.97)	0.93 (0.91- 0.94)	0.91 (0.90- 0.93)	0.95 (0.94‐0.95) (*P*=.13)^c^	0.97 (0.96-0.98) (*P*=.49)	0.95 (0.93‐0.96) (*P*=.25)	0.94 (0.92‐0.95) (*P*=.28)
Dense breast group (density C and D)	0.82 (0.81‐0.83)	0.88 (0.86‐0.90)	0.81 (0.79‐0.83)	0.81 (0.79- 0.82)	0.84 (0.83‐0.85) (*P*=.20)	0.88 (0.86‐0.90) (*P*=.90)	0.85 (0.87‐0.93) (*P*=.22)	0.82 (0.81‐0.83) (*P*=.59)

a AUROC: area under the curve of the receiver operating characteristic.

b AI: artificial intelligence.

c Represents *P* value of <.01 for paired groups by reader experience before and after AI assistance.

### Agreement Rate Between Radiologists and AI

The overall agreement rate between consultants, senior radiologists, and junior radiologists with AI was 84.1%, 81.3%, and 81.4%, respectively (Table 3). The agreement rate among junior residents before and after AI assistance was κ=0.54 to κ=0.60, *P*=.08. The agreement rate among senior residents before and after AI assistance was κ=0.59 to κ=0.62 *P*=.48. The agreement rate among consultants before and after AI assistance was 0.66 to 0.71, *P*=.07.

**Table 3.** Agreement rate between AI^a^ and radiologists.

	Consultants	Senior residents	Junior residents
Agreement rate between AI and radiologists (% proportion)	84.1	81.0	81.4
Discordance			
Total number of readings across scenarios, (number of patients)	319 (145)	380 (176)	838 (220)
Scenario 1			
AI is correct, reader false negative, number of cases (% proportion of discordant cases)	157 (49.2)	215 (56.6)	502 (59.9)
Scenario 2			
AI is correct, reader false positive, number of cases (% proportion of discordant cases)	17 (5.3)	23 (6.1)	58 (6.9)
Scenario 3			
Reader correct, AI false negative, number of cases (% proportion of discordant cases)	66 (20.7)	60 (15.8)	97 (11.6)
Scenario 4			
Reader correct, AI false positive, number of cases (% proportion of discordant cases)	79 (24.8)	82 (21.6)	181 (21.6)

a AI: artificial intelligence.

### Reading Time Analysis

For all radiologist groups, significant time savings were achieved for both non-malignant and malignant cases following AI assistance, with the highest time savings observed in non-malignant cases (18.0 [SD 34.3] s (95% CI [17.0, 19.0]) vs malignant cases (11.1 [SD 34.2] s (95% CI [10.1, 12.1] per mammogram read after AI assistance, *P*<.001). Time savings were highest for the junior residents, followed by the consultants and the senior residents (Table 4).

**Table 4.** Time savings per mammogram read for radiologist groups with and without AI assistance.

	Avg.^a^ time per mammogram read without AI in seconds, mean (SD)^b^			Avg. time per mammogram read with AI in seconds, mean (SD)			Avg. time savings per mammogram read with AI in seconds, mean (SD)		
	Consultant	Senior Resident	Junior resident	Consultant	Senior Resident	Junior resident	Consultant Time savings, 95% CI of difference	Senior Resident Time savings, 95% CI of difference	Junior resident Time savings, 95% CI of difference
All cases	81.0 (36.2)	58.2 (27.2)	70.0 (41.9)	70.2 (31.5)	50.1 (22.8)	50.1 (29.1)	10.4 (34.0) (95% CI 8.6‐11.6), *P*<.001	8.1 (27.5) (95% CI 6.9‐9.3), *P*<.001	19.7 (36.8) (95% CI 18.3‐20.5), *P*<.001
Non-malignant cases	73.1 (35.1)	55.5 (26.3)	66.7 (42.4)	57.5 (26.2)	44.6 (18.9)	43.4 (28.2)	15.8 (31.8) (95% CI 12.9‐17.0), *P*<0.001	11.1 (25.3) (95% CI 9.0‐12.2), *P*<.001	23.4 (37.7) (95% CI 21.1‐24.2), *P*<.001
Malignant cases	88.1 (35.8)	60.0 (27.7)	73.0 (41.2)	83.0 (31.2)	55.7 (25.0)	56.7 (28.4)	4.9 (33.9) (95% CI 3.1‐7.3), *P*<.001	5.5 (26.7) (95% CI 3.8‐7.3), *P*<.001	15.4 (36.6) (95% CI 14.7‐17.7), *P*<.001

a Avg.: average.

b SD: standard deviation.

## Discussion

### Principal Findings and Comparison With Previous Works

This study marks the first exploration of AI assistance for resident radiologists in Asia. The tested AI software exhibited a high overall AUROC performance of 0.93, aligning with results of prior research in western populations by Aggarwal et al [24] and Hickman et al [25], achieving AUROCs of 0.87 and 0.89, respectively. Our study demonstrated that AI assistance improved diagnostic performance across all radiologist groups, with the greatest relative gains observed among residents. While the AUROC for senior residents using AI approached that of consultants (0.88 vs 0.90, *P*=.051), this trend suggests the potential of AI to support less experienced readers and help narrow the performance gap between intermediate and expert radiologists, although the difference did not reach statistical significance. At least two-thirds of women in our study had high breast density. Dense breasts are known to increase the difficulty of mammographic interpretation, which is a particular challenge in Asian populations, where breast density tends to be higher compared to Western counterparts [2,26]. With AI assistance, the cancer detection rate on mammograms of women with high breast density showed improvement across all radiologist groups, with senior residents experiencing the highest improvement, reflected in an AUROC increase from 81% to 85%. The ability of any AI software to deliver diagnostic performance comparable to that of a second reader in double-reading systems introduces the potential to streamline workflows, reducing the reliance on a second reader and alleviating radiologists’ workload. However, to fully realize this potential, rigorous testing of various thresholds and continuous evaluations with feedback loops will be necessary. These measures will ensure the system evolves over time and remains aligned with clinical outcomes, maintaining its utility and effectiveness in real-world settings [27].

Furthermore, AI assistance was shown to improve sensitivity and accuracy for radiologists at all levels, particularly benefiting junior and senior residents. This supports the idea that AI can help mitigate the effects of lower diagnostic experience, which is commonly seen in radiology residents who have not yet accrued a high volume of cases. The study by Agarwal et al [28] underscores the correlation between a resident’s diagnostic performance and the number of studies they interpret during their training, making the assistance of AI a valuable asset in overcoming this learning curve. For consultant radiologists, the gains in accuracy were less pronounced, suggesting that the advantage of AI assistance is more substantial for those earlier in their careers. This phenomenon might be explained by the “regression to the mean” effect, where extreme performances (eg, by junior radiologists with less experience) tend to improve with intervention (AI assistance), while those with already higher baseline performance (experienced consultants) see more modest improvements [29]. While AI enhances overall diagnostic capabilities, its transformative impact is most evident at lower experience levels, where there is more room for improvement.

Beyond individual benefits, incorporating AI into structured residency curricula focused on practical applications offers an opportunity to enhance diagnostic proficiency, accelerate skill acquisition, and foster confidence in early-career clinicians. This preparation is essential for adapting to the rapidly evolving and digitizing field of medical imaging [30]. A nationwide cross-sectional survey by Chen et al, involving 3666 participants, revealed that radiology residents generally hold positive attitudes toward AI, with 72.8% acknowledging its potential to improve disease diagnosis and 78.18% emphasizing the importance of embracing AI in practice. However, 29.9% expressed concerns about AI reducing the demand for radiologists. Maria et al [31] described the feasibility of a 3-day AI curriculum that successfully improved radiologists’ perceptions of their knowledge and skills regarding AI in radiology, serving as a potential model for further adaptation and implementation in educational settings.

The observation that both junior and senior radiologists demonstrated lower sensitivity but higher specificity compared to the AI software underscores key differences in diagnostic strategies between human radiologists and AI systems. With AI assistance, however, both groups showed a significant improvement in sensitivity without sacrificing specificity. This suggests that radiologists may naturally prioritize specificity to avoid false positives and unnecessary interventions, which explains their higher specificity. However, this cautious approach can lead to reduced sensitivity, potentially resulting in missed diagnoses (false negatives), particularly in subtle or complex cases. In contrast, AI systems are typically designed to maintain high sensitivity thresholds, leveraging their capability to identify subtle abnormalities that might be overlooked by human readers. This distinction highlights the complementary strengths of human radiologists and AI [32]. Hence, integrating AI into clinical workflows may help achieve a better balance, enhancing radiologists’ sensitivity without compromising specificity, ultimately improving diagnostic accuracy. Enhanced sensitivity through AI integration can help reduce false negatives, leading to earlier detection and treatment of conditions like breast cancer. At the same time, maintaining high specificity reduces unnecessary biopsies, alleviating patient discomfort and lowering health care costs [33].

Next, AI assistance was found to improve efficiency, particularly through time savings, with longer savings observed in non-malignant cases. This indicates that the AI software could offer substantial cost savings by enhancing efficiency, especially in screening settings where the ratio of non-cancer to cancer cases is typically higher [34]. Consistent with cost-effectiveness analyses of AI solutions in other domains, the greatest time savings were seen in non-specialist groups [35] rather than expert radiologists, where the marginal advantages are limited. Furthermore, AI assistance improved inter-reader agreement across all radiologist groups, with junior residents benefiting the most. This highlights AI’s potential to reduce variability in diagnostic decisions and enhance consistency among radiologists of varying experience levels. However, the lack of perfect agreement between radiologists and AI indicates that discrepancies in interpretation persist. These disagreements often lead to additional time spent analyzing differences, further investigations, or the need for arbitration by additional personnel. Mehrizi et al [36] observed that when AI provided an incorrect suggestion but radiologists ultimately made the correct decision, these tasks took longer, averaging 89 seconds compared to the overall average of 79 seconds. Therefore, the thoughtful integration of AI into clinical workflows is important to ensure that its benefits, such as improved efficiency and consistency, outweigh any added complexities or challenges.

The strength of this study lies in its provision of valuable insights into how AI can complement human decision-making and enhance diagnostic performance at different stages of professional development. Nonetheless, several limitations should be acknowledged. First, the dataset was enriched to a 50% cancer prevalence, consistent with previous multi-reader studies [3,4], to reduce the total number of cases required and minimize the burden on the 17 participating readers while retaining the ability to assess diagnostic accuracy. However, as real-world screening populations typically have lower cancer prevalence, there is concern that the AI system may exhibit a higher false positive rate in clinical practice. In addition, the lack of clinical variables, such as patient age, prior imaging, and treatment history, may limit the model’s robustness and reduce its generalizability across diverse patient populations. While the study was not specifically powered for formal hypothesis testing across all reader subgroups, the inclusion of 4 consultants, 4 senior residents, and 9 junior residents exceeds the minimum threshold recommended by Hillis and Schartz [11] for generalizing to both reader and case populations. This permitted exploratory analyses of variability in diagnostic performance by reader experience level. Due to logistical constraints, a crossover design was not implemented, introducing the possibility of unintended training effects, where improved reading efficiency during the second round may have been due in part to increased familiarity rather than the AI intervention. To mitigate this, several strategies were used: a pilot session was conducted to familiarize readers with the AI tool; case order was shuffled between rounds; and a washout period was introduced. These measures were designed to minimize practice effects and enhance the attribution of performance changes to the AI tool itself.

Despite these limitations, this study remains a crucial initial step in assessing the performance of AI software in supporting resident radiologists, as well as in the context of dense Asian breasts. The study process revealed insights into potential challenges that could emerge during the initial phases of integrating AI [37], such as the need for interpretive adjustment and the issue of misdiagnosis, which requires careful consideration. Clear definitions of the roles and accountabilities of AI, senior residents, and consultants in the event of misdiagnosis are essential [38]. Future endeavors will include an in-depth analysis of the discordance cases between radiologist groups and the AI solution, understanding perspectives of human readers and establishing a feedback loop for model improvement to ensure that AI becomes a valuable tool in health care while maintaining patient safety and quality of care. Lastly, a prospective multi-center collaboration would provide a larger and more diverse dataset, representative of the Asian population, to assess the AI system’s effectiveness across a broader range of cases and demographics [39].

#### Conclusions

In summary, this study provides preliminary insights into the use of AI assistance in mammographic interpretation among radiologists in Singapore. While AI showed some potential in improving sensitivity, particularly for junior and senior residents, its overall impact on diagnostic accuracy was modest, with many comparisons not reaching statistical significance. Automation bias and inter-reader variability remain challenges that require careful consideration. The enriched dataset and absence of a crossover design are also some of the limitations of our study. Future studies should prioritize multi-center collaborations, external validation, and in-depth analyses of discordant cases to ensure AI integration enhances diagnostic workflows while maintaining safety and clinical relevance.

## Supplementary material

10.2196/66931 Checklist 1 STARD checklist.

## Abbreviations

AI: artificial intelligence
AUROC: area under curve of the receiver operating characteristic
BIRADS: Breast Imaging Reporting and Data System
MRMC: multi-reader multi-case
NUH: National University Hospital
